# Patterns in the Economic Burden of Acute Kidney Injury in Hospitalized Children, 2019-2021

**DOI:** 10.1001/jamanetworkopen.2023.17032

**Published:** 2023-06-05

**Authors:** Rupesh Raina, Anvitha Soundararajan, Natalie Menassa, Aadi Pandya, Carla Nemer, Abhishek Tibrewal, Sidharth Kumar Sethi

**Affiliations:** 1 Department of Medicine, Northeast Ohio Medical University, Rootstown, Ohio; 2 Department of Pediatric Nephrology, Akron Children’s Hospital, Akron, Ohio; 3 Akron Nephrology Associates, Cleveland Clinic Akron General Medical Center, Akron, Ohio; 4 Northeast Ohio Medical University, Rootstown, Ohio; 5 Pediatric Nephrology, Kidney Institute, Medanta, The Medicity, Gurgaon, India

## Abstract

This economic evaluation uses data from the Pediatric Health Information System to assess patterns in the economic burden of acute kidney injury and variables correlated with mortality and length of stay among hospitalized US children with acute kidney injury between 2019 and 2021.

## Introduction

Pediatric acute kidney injury (AKI) is common, especially in hospitalized children, its incidence being 18.7% overall and 24.4% among critically ill patients.^1^ Children with AKI have increased odds of higher length of stay (LOS), death, hypertension, proteinuria, and chronic kidney disease.^1,2^ Development of AKI increases health care costs and resource use burden. Despite the increasing incidence of pediatric AKI,^1^ few studies have evaluated its US economic burden. We used the Pediatric Health Information System (PHIS) to assess the financial burden associated with AKI. The PHIS provides data on treatment costs for pediatric diseases, which were used to evaluate patterns in the economic burden of AKI and variables correlated with mortality and LOS among hospitalized children with AKI.

## Methods

This economic evaluation was approved by the Akron Children’s Hospital Institutional Review Board. Patient consent was waived because all data were deidentified. The study followed the Consolidated Health Economic Evaluation Reporting Standards (CHEERS) reporting guideline.

We included participants younger than 18 years who were discharged from participating hospitals between April 1, 2019, and January 31, 2021, with a diagnosis of AKI (eMethods in Supplement 1). The variables considered per AKI case were children’s hospitals case mix index (CHCMI), percentage of major complications or comorbidity (MCC), number of *International Classification of Diseases, Tenth Revision* diagnosis and procedure codes, mortality rate, LOS, and adjusted charges. The CHCMI was calculated by dividing mean hospitalization cost for AKI diagnoses recorded in the Healthcare Cost and Utilization Project (HCUP) database and the Kids’ Inpatient Database (KID) by mean hospitalization cost of all hospitalizations at a children’s hospital recorded in HCUP or KID. Pearson correlation and multivariate regression analyses were conducted. Two-sided *P* < .05 was considered significant. Data were analyzed using IBM SPSS Statistics, version 22.

## Results

A total of 6797 children with AKI from 49 hospitals were included. The mean (range) number of diagnosis and procedure codes per AKI case was 11.7 (10.2-13.8) and 1.2 (0.8-1.6), respectively. Overall, 2756 children (40.1%) had MCC (Table 1). The mean (range) CHCMI was 1.08 (0.95-1.21), representing 8% higher relative cost of AKI vs typical hospitalization. The mortality rate was 0.44% (299 children), the mean (range) LOS was 6.2 (5.2-7.2) days, and the mean (range) adjusted charges were $72 460 ($51 060-$93 860) across all hospitals. Correlations of variables with mortality and LOS are shown in Table 2. None of the variables were associated with mortality in a multivariate logistic regression model. Higher mortality was correlated with LOS (*r* = 0.432 [95% CI, 0.171-0.636]; *P* = .002); higher LOS was correlated with adjusted charges (*r* = 0.574 [95% CI, 0.350-0.737]; *P* < .001).

**Table 1.** Summary of Variables Assessed Across 49 Hospitals With Pediatric Acute Kidney Injury Cases

Variable	Weighted mean (range)
CHCMI	1.08 (0.95-1.21)
MCC cases of total AKI cases, %	40.1
No. of diagnosis codes per AKI case	11.7 (10.2-13.8)
No. of procedure codes per AKI case	1.2 (0.8-1.6)
Mortality rate, %	0.44
Length of stay, d	6.2 (5.2-7.2)
Adjusted charges per AKI case, $	72 460 (51 060-93 860)

Abbreviations: AKI, acute kidney injury; CHCMI, children’s hospitals case mix index; MCC, major complications or comorbidity.

**Table 2.** Correlation of Variables With Mortality Rate and Length of Stay

Variable	Mortality rate		Length of stay	
	Pearson *r* (95% CI)	*P* value	Pearson *r* (95% CI)	*P* value
CHCMI	0.375 (0.105-0.594)	.008	0.596 (0.378-0.751)	<.001
MCC cases of total AKI cases	0.372 (0.102-0.591)	.008	0.517 (0.276-0.697)	<.001
No. of diagnosis codes per AKI case	NA	NA	0.402 (0.136-0.614)	.004
No. of procedure codes per AKI case	0.410 (0.146-0.620)	.003	0.647 (0.448-0.786)	<.001
Mortality rate	NA	NA	0.432 (0.171-0.636)	.002
Length of stay	0.432 (0.171-0.636)	.002	NA	NA

Abbreviations: AKI, acute kidney injury; CHCMI, children’s hospitals case mix index; MCC, major complications or comorbidity; NA, not applicable.

## Discussion

This economic evaluation of PHIS data found adjusted charges of $72 460 per AKI case. Pediatric AKI was associated with 8% higher hospitalization cost vs routine hospitalization, with key factors being LOS, number of procedure and diagnosis codes, and CHCMI. These strategies could be facilitated using electronic health record–enabled informatics, such as AKI prediction models, automated real-time AKI alerts, and tracking of patients over time and across institutions, registries, and databases.^3^ The physician could be automatically notified, and orders for creatinine and albumin to creatinine ratios could be generated at discharge for timely assessment.^3^ This study’s limitations relate to use of the PHIS, an administrative database that lacks specific clinical information and is limited to single care episodes, thus accounting for only part of the total costs.

More cost-effective treatment strategies are needed.^4^ Initiatives such as the Saving Young Lives project help prevent AKI by alerting, educating, and providing resources to clinicians.^5^ Our findings are a call to action for hospitals, policy makers, researchers, and pharmaceutical companies to acknowledge and address the financial burden of pediatric AKI.

## Notes

Supplement 1. **eMethods.** Study Design, Study Population, Measurements, Outcome, and Statistical Analysis
eReferences

Supplement 2.
Data Sharing Statement
