# Supplementary material for: Patterns in the Economic Burden of Acute Kidney Injury in Hospitalized Children, 2019-2021
Source: JAMA Netw Open. 2023 Jun 5;6(6):e2317032. doi: 10.1001/jamanetworkopen.2023.17032 (PMC10242423; doi:10.1001/jamanetworkopen.2023.17032)
Supplement: Supplement 1. — eMethods. Study Design, Study Population, Measurements, Outcome, and Statistical Analysis eReferences [file jamanetwopen-e2317032-s001.pdf]

Supplementary Online Content

Raina R, Soundararajan A, Menassa N, et al. Patterns in the economic burden of acute kidney injury in hospitalized children, 2019-2021. *JAMA Netw Open*. 2023;6(6):e2317032. doi:10.1001/jamanetworkopen.2023.17032

eMethods. Study Design, Study Population, Measurements, Outcome, and Statistical Analysis

eReferences

This supplemental material has been provided by the authors to give readers additional information about their work.

© 2023 Raina R et al. *JAMA Network Open*.

eMethods. Study Design, Study Population, Measurements, Outcome, and Statistical Analysis

Study Design

This study is a retrospective analysis of the Pediatric Health Information System database. The PHIS database was utilized to investigate trends in increased cost in AKI. It is a comparative pediatric database with clinical and resource utilization data for inpatient, ambulatory surgery, emergency department, and observation unit patient encounters for more than 49 freestanding children's hospitals across different US cities[1]. These hospitals are affiliated with the Children's Hospital Association (Lenexa, Kansas). Data quality and reliability are assured through a joint effort between the Children's Hospital Association and participating hospitals. Data is deidentified at the time of submission and is subjected to several reliability and validity checks before inclusion in the database[1].Institutional Review Board approval was obtained for our own institutional review of our PHIS data related to this analysis. Patient consent was waived as all data was deidentified.

Study population

Inclusion criteria: All inpatients younger than 18 years who were discharged from a participating hospital between April 1, 2019, and January 31, 2021 with diagnosis of AKI, based on APR-DRG code 4692. The severity of AKI and the number of children with or without dialysis were not specified in the database.

Exclusion criteria: We excluded adult patients (aged ≥18 years)

Measurements

Among children with AKI admitted across PHIS hospitals, the variables considered were as follows: children's hospital case mix index (CHCMI), percent with major complication or comorbidity (MCC), number of ICD 10 diagnosis codes per AKI case, number of procedure codes per AKI case, percent with mortality, length of stay (LOS) for children with AKI, adjusted

© 2023 Raina R et al. *JAMA Network Open*.

charges per AKI case. Children Hospital Case Mix Index is the cost of AKI hospitalization relative to the cost of a ‘typical’ hospitalization at a Children Hospital (CH). This measure compares the severity of illness between patients at various hospitals. Utilizing the Healthcare Cost and Utilization Project's (HCUP) collection of databases, which includes the Kids' Inpatient Database (KID), CMI is calculated by dividing the mean hospitalization cost for diagnoses of AKI at a CH in HCUP KID by the mean hospitalization cost of all hospitalizations at a CH in HCUP KID. MCC is a metric that depicts diagnoses that occur as a result of AKI that always require additional interventions and have a large impact on a patient’s stay. ICD 10 and procedure codes are useful analytic metrics that provide further classification of individual patient cases and the unique complexities associated with each, such as varying modalities of dialysis which can influence cost factor. The database did not specify the different major complication or comorbidity that were included as part of MCC. Also, no description was provided about the different ICD 10 diagnosis codes or procedures that were reported among the children with AKI. Moreover, the different components that were included for the calculation of adjusted charges per AKI case were not specified in the database.

Outcome

The outcomes included a) Mortality rate, b) Average length of stay and c) Adjusted charges per cases for AKI cases across different hospital cities as well as across PHIS overall.

Statistical Analysis

Microsoft Excel was used to record all variables. The statistical analyses were performed using statistical software (SPSS version 22). The Kolmogorov-Smirnov test was used to determine the normality of all the variables. The continuous variables are reported as the weighted average and the categorical values as percentages (the sum of weights times values

© 2023 Raina R et al. *JAMA Network Open*.

divided by the sum of the weights). For each variable, the medians and interquartile range (IQR; 25th to 75th percentiles) for each hospital site have also been reported. The Pearson correlation coefficient was used to examine the relationship between the variables. A correlation and multivariate linear regression analysis was conducted and a two-sided p value <0.05 was considered statistically significant.

© 2023 Raina R et al. *JAMA Network Open*.

eReferences

1. Leverage Clinical and Resource Utilization Data. Children's Hospital Association. https://www.childrenshospitals.org/content/analytics/product-program/pediatric-health-information-system
2. 3M™ all patient refined diagnosis related groups (APR DRG) methodology ... https://medicaid.ohio.gov/static/Providers/ProviderTypes/Hospital/APRDRGV34.pdf. Accessed January 14, 2023.

© 2023 Raina R et al. *JAMA Network Open*.
