# Supplementary material for: Patterns in the Economic Burden of Acute Kidney Injury in Hospitalized Children, 2019-2021
Source: JAMA Netw Open. 2023 Jun 5;6(6):e2317032. doi: 10.1001/jamanetworkopen.2023.17032 (PMC10242423; doi:10.1001/jamanetworkopen.2023.17032)
Supplement: Supplement 2. — Data Sharing Statement [file jamanetwopen-e2317032-s002.pdf]

Data Sharing Statement

Raina. Patterns in the Economic Burden of Acute Kidney Injury in Hospitalized Children, 2019-2021. *JAMA Netw Open*. Published June 05, 2023. doi:10.1001/jamanetworkopen.2023.17032

Data
Data available: Yes
Data types: Deidentified participant data
How to access data: Data will be made available by contacting the corresponding author, Dr.Rupesh Raina at rraina@akronchildrens.org
When available: With publication

Supporting Documents
Document types: None

Additional Information
Who can access the data: Data will be made available to anyone requesting the data.
Types of analyses: For any purpose.
Mechanisms of data availability: With a signed data access agreement.
